# The efficacy of low/lactose-free milk powder in the treatment of lactose intolerance in infants: A protocol for systematic review and meta analysis

**DOI:** 10.1097/MD.0000000000039098

**Published:** 2024-08-02

**Authors:** Kalibinuer Aierken, Zhiwei Xu, Jianbao Ma, Gulibahaer Kawuli

**Affiliations:** a Department of Pharmaceutics and Physical Chemistry, College of Pharmacy, Xinjiang Medical University, Urumqi, China.

**Keywords:** diarrhea in children, lactose intolerance, meta-analysis

## Abstract

**Background:**

The aim of this study was to evaluate the efficacy and safety of formula milk powder in the treatment of lactose intolerance in children, and to provide an evidence-based medicine basis for the rational use of drugs in children with lactose intolerance caused by various reasons by meta-analysis.

**Methods:**

Use computers to search major databases, including Web of Science, PubMed, CNKI, Wanfang Data Knowledge Service Platform, and other databases, the retrieval time is from the establishment of the database to April 2023. The collected literatures were screened, data extracted and processed, and then meta-analysis was performed by Review-Manager 5.4 statistical software.

**Results:**

A total of 10 randomized controlled trials were included, with 1112 patients, including 562 patients in the treatment group and 550 patients in the control group. The control group was treated with conventional therapy, and the treatment group was treated with lactose-free/low-lactose milk powder on the basis of conventional therapy. The results of the meta-analysis showed that the clinical efficacy of the treatment group was significantly better than that of the control group [odds ratio=6.01, 95% confidence interval (CI): 3.94–9.18, *P*<0.00001], the course of disease in the treatment group was shorter than that in the control group (mean difference=−1.45, 95% CI: −1.76 to −1.13, *P*<0.0001). The antidiarrhea time of the treatment group was shorter than that of the control group, and the difference between the 2 groups was statistically significant (mean difference=−1.41, 95% CI: −1.67 to −1.15, *P*<0.0001).

**Conclusion:**

Low/lactose-free milk powder can improve clinical efficacy and shorten the course of treatment in infants with lactose intolerance, which can be demonstrated by further large-scale clinical studies.

## 1. Introduction

Diarrhea is the most common cause of death in children under 5 years old in China. It is a digestive tract syndrome caused by multiple pathogens and factors, characterized by increased stool frequency and changes in stool characteristics. Diarrhea is one of the main causes of malnutrition and growth disorders in children, and is a global public health problem.^[1–3]^ The clinical manifestations of diarrhea caused by virus in children are vomiting, abdominal pain, diarrhea, and other digestive tract symptoms, which can lead to chronic diarrhea, dystrophy, rickets, weight and mental retardation, growth retardation and other symptoms, and can damage the small intestinal villi, and then cause lactase destruction, causing the body to absorb lactose disorders, called (secondary lactose intolerance, SLI); lactase deficiency in humans is a key factor in the symptoms of lactose intolerance (lactose intolerance, LI).^[4]^ Lactose is the most abundant sugar in animal milk (including human breast milk) and modified cow’s milk formula. If young children are unable to digest and absorb lactose due to lactase deficiency, continued intake of lactose-containing milk or milk products may exacerbate acute diarrhea due to osmotic effect. This may exacerbate dehydration, malabsorption, malnutrition, and growth disorders, and may eventually lead to death of children suffer from severe diarrhea in areas of the world where severe diarrhea is common.^[5]^

At present, for the treatment of acute diarrhea caused by viruses, there are more conventional treatments plus a complementary therapy to shorten the course of disease and improve the prognosis. Wang,^[6]^ using Shenling powder with probiotics to treat acute diarrhea, the effect is improved than the conventional therapy; Lin,^[7]^ the use of Live Combined Bifidobacterium and Lactobacillus Tablets combined with montmorillonite powder in the treatment of acute diarrhea has also improved the effect. However, due to the particularity of infants and young children, there is more or less risk of combination therapy, Du,^[8]^ it is reported that severe allergic reaction of children caused by taking Shenling Baizhu San. Food-based adjuvant therapy is safer and more reliable than medicine. Therefore, more attention should be paid to low/lactose-free diet adjuvant therapy for acute diarrhea in children!

According to the database data, there is no systematic review on the effectiveness of low/lactose-free milk powder in the treatment of infant lactose intolerance, therefore, this paper uses the meta-analysis method to explore the beneficial effect of low/lactose-free milk powder in improving the symptoms of lactose intolerance in infants and young children, and provide reference for clinical application.

## 2. Data and methods

### 2.1. Ethical approval

This study is a systematic review, the research object is literature, and does not involve ethical issues.

### 2.2. Literature search

Literature search was conducted in domestic and foreign databases, including China national knowledge infrastructure (CNKI), Wanfang, Weipu, Pubmed, Springer Link, Web of Science, supplemented by manual search. Chinese search terms included: 腹泻, 乳糖酶, 乳糖不耐受, 随机对照实验 (Randomized Controlled Trial, RCT). English search terms include: lactose intolerance, diarrhea, low-lactose milk powder, and clinical randomized controlled studies. The search period is from self-built database to February 2023. Through the initial literature search, as well as more relevant literature survey, first included in the title of the literature, for its abstract screening, in the relevant systematic review or meta-analysis to meet the inclusion criteria by reading the references to expand the scope of the search, found that other studies are also included.

### 2.3. Literature inclusion and exclusion criteria

#### 2.3.1. Inclusion criteria

Study population: International and domestic published literature on lactose intolerance in children, the reference language is limited to English and Chinese, gender, race is not limited. The control group and the experimental group were treated with conventional treatment and breast milk/lactose-free milk powder/lactose-free milk powder. Outcome measures: The main outcome measures were effective rate, course of disease, time of remission and disappearance of diarrhea symptoms and time of disappearance of vomiting. Type of study: RCT. Because the abstract does not provide complete relevant information, only the full-text data is adopted;

#### 2.3.2. Exclusion criteria

① Studies with out-of-scope outcome indicators; ② poor quality: literatures with incomplete data and repeated reports; ③ case reports, animal experiments and reviews.

### 2.4. Literature extraction

#### 2.4.1. Literature screening and data extraction

Two investigators independently searched and screened the literature according to the inclusion and exclusion criteria, evaluated the quality of the literature, and decided whether to include it or not when there was disagreement. Data extraction was also independently completed by 2 investigators, and the final decision was made by the third party in case of disagreement. The extraction included the first author, publication year, country of origin of cases, total number of included cases, number of grouped cases, treatment plan intervention measures, outcome indicators, etc.

### 2.5. Quality assessment

Two investigators independently assessed the quality of the included RCTs according to the Cochrane Guidelines for Systematic Review. Disagreement was assessed by a third party. There were 6 criteria, each of which was divided into “1,” “−1,” and “0.” “1” means low risk of bias, “−1” means high risk of bias, and “0” means uncertain risk of bias.

### 2.6. Statistical methods

Meta-analysis was performed with Review-Manager 5.4 statistical software. Odds ratio (OR) was used as effect size for dichotomous variables and mean difference (MD) was used as effect size for continuous variables. The effect size was expressed with 95% confidence interval (95%CI). Heterogeneity test was performed for the included studies. If *P* > .1, *I*^2^ < 50%, it was considered that there was still controversy among the studies and there was no statistical heterogeneity among the studies. If *P* < .1, *I*^2^ > 50%, the study was considered statistically heterogeneous, using random effects model analysis.

## 3. Results

### 3.1. Literature search results

**Figure 1.**
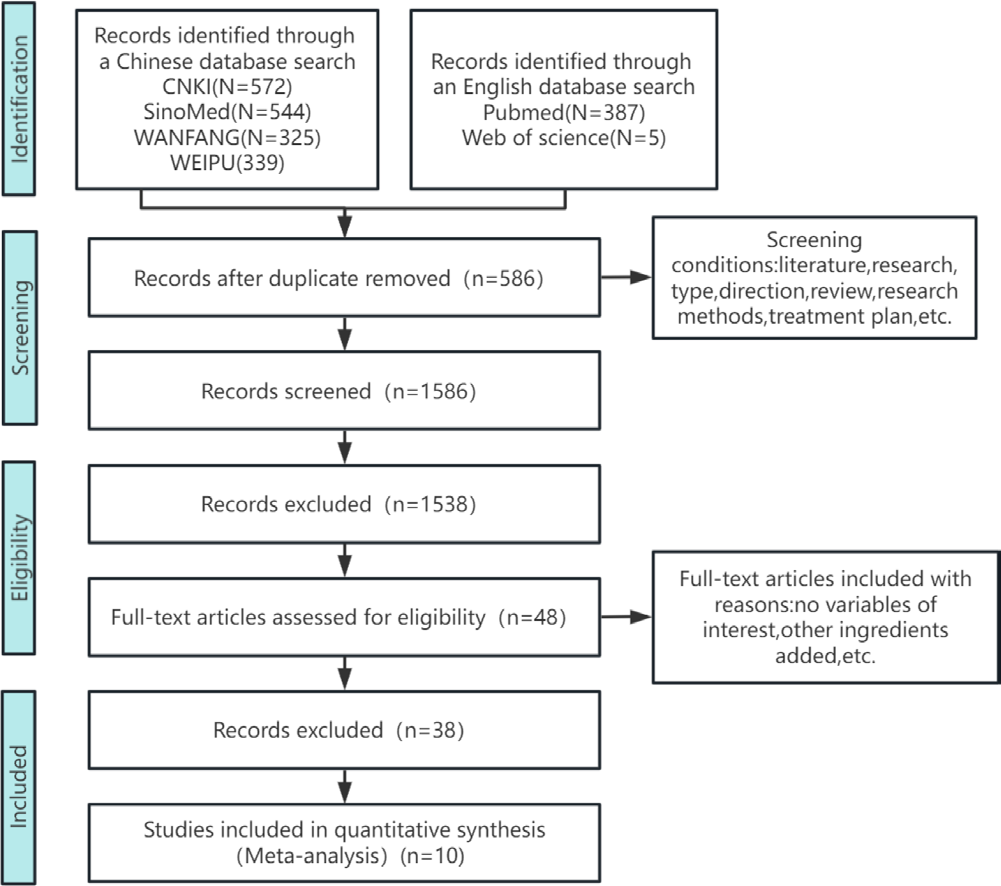
Flow chart of literature inclusion.

A total of 2172 articles were initially decorated according to key words, 2124 articles that were repetitive and obviously did not meet the inclusion criteria after reading the title and abstract were excluded, and the articles with unqualified intervention objects, unqualified intervention measures and incomplete outcome indicators were further excluded after reading the full text. Finally, 10 articles^[6–15]^ were included, all of which were RCT studies, all of which were Chinese literatures, with a total of 1112 patients, including 562 patients in the treatment group and 550 patients in the control group. See Figure 1 for the literature inclusion process. See Table 1 for the basic data characteristics of the inclusion studies.

**Table 1:** Basic information on the included literature.

Basic data							Intervention program	Intervention time (d)	Outcome measures
Included studies	Disease condition	Number of cases	Group		Gender (M/F)	Age (mo)			
Li ^[9]^	Infantile diarrhea	210	control group	105	69/36	5–18 (12.5 + 5.8)	The control group continued to breastfeed	3	①②
			Treatment group	105	67/38		Feeding with sugar-free milk powder		
Zhu et al^[10]^	Infantile diarrhea	60	Control group	30	17/13	6–12	Routine culture in control group	3	①
			Treatment group	30	14/16		The treatment group was treated with routine therapy and low-lactose milk powder		
Li^[11]^	Infantile diarrhea	96	Control group	48	57/39	5–24 (10.40 ± 2.72)	The control group was fed with long-term lactose-free milk powder	3–5	①③④⑤
			Treatment group	48			The observation group was fed with short-term lactose-free milk powder		
Wang^[12]^	Infantile diarrhea	96	Treatment group	48	26/22	6–36	On the basis of routine treatment, the observation group was fed with lactose-free formula milk powder	3	①②③
			Control group	48	26/22	7–36	On the basis of conventional treatment, the control group was given breast-feeding or common formula feeding		
Xia et al^[13]^	Infantile diarrhea	111	Treatment group	56	35/21	(12.58 ± 5.48)	Under conventional treatment, stop lactose-containing milk powder or breast-feeding, and use lactose-free milk powder	2–8	①②③
			Control group	55	37/18	(12.88–5.12)	Continue breast milk or formula under conventional treatment		
Xu and Huang^[14]^	Infantile diarrhea	126	Treatment group	63	75/51	≤12	Under conventional treatment, supplemented with low-lactose milk powder feeding	7	①②③
			Control group	63			Under conventional treatment, supplemented by conventional milk powder feeding		
Hu et al^[15]^	Infantile diarrhea	124	Treatment group	60	33/27	2.6–21.2	On the basis of conventional treatment, low-lactose milk powder feeding	3	①
			Control group	64	33/31	2.9–20.3	On the basis of routine treatment, supplemented by the original feeding		
Zhang^[16]^	Infantile diarrhea	80	Treatment group	42	23/19	3.3–25	On the basis of routine treatment, low-lactose milk powder was added as an adjuvant treatment	3	①⑥⑦
			Control group	38	20/18	3–26	Conventional treatment		
Zhou and Wang^[17]^	Infantile diarrhea	119	Treatment group	65	75/44	5–24	Conventional treatment plus weaning	3	①③⑥
			Control group	54			Conventional treatment plus breast-feeding		
Wang^[18]^	Infantile diarrhea	90	Treatment group	45	/	≤18	Conventional treatment plus lactose-free feeding	3	①②
			Control group	45			Conventional treatment plus breast feeding		

Outcome indicators: ① effective rate; Total course of disease; ② Total course of disease; ③ The time for the disappearance of vomiting; ④ Body weight increment ⑤ Respiratory tract infection rate; The time for the disappearance of vomiting.

F = Female, M = Male.

### 3.2. Literature quality evaluation and risk of bias

**Figure 2.**
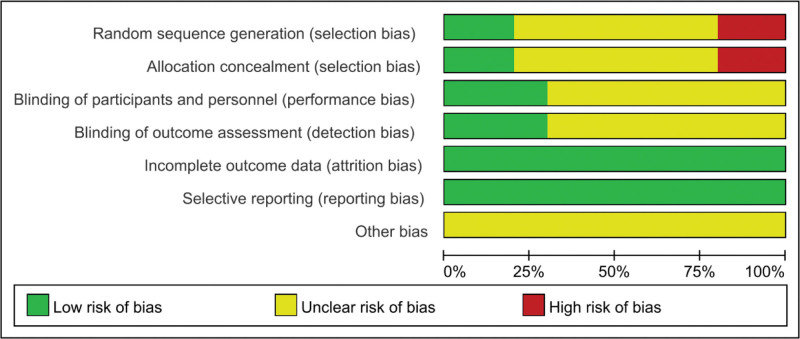
Risk of bias map for inclusion in the literature.

**Figure 3.**
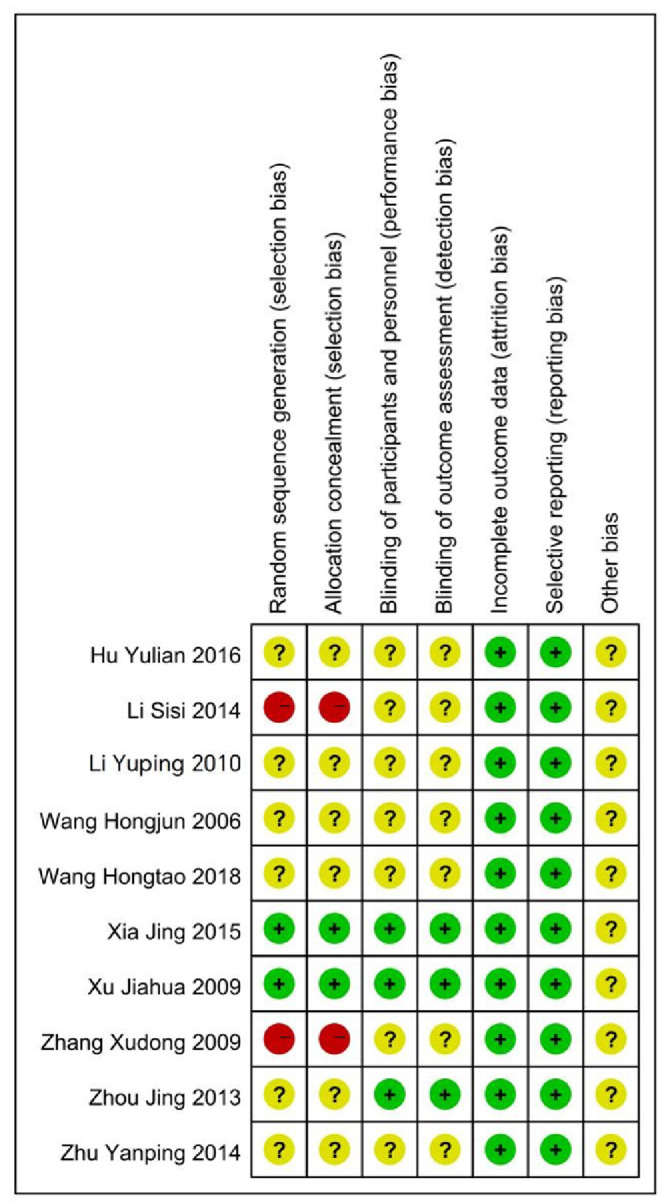
Summary of risk of bias in the inclusion literature.

The quality of included RCTs was evaluated according to the Cochrane Guidelines for Systematic Reviews. All included studies had certain risks. In 2 studies, the risk of random sequence generation and allocation concealment bias was high. There were 6 studies with incomplete information on allocation concealment, random sequence, data integrity, selective reporting, etc. (The quality evaluation of the specific included literature is shown in the Table 2; Figs. 2 and 3) .

**Table 2:** Summary of randomized controlled trial bias risks.

Documentation (literature)	Random sequence generation (randomization)	Allocation hiding (distribution scheme hiding)	Blind method (blinding)	Data integrity (data integrity)	Selective report (selective report result)	Other (other bias)
Li ^[9]^	0	0	0	1	1	0
Zhu et al ^[10]^	0	0	0	1	1	0
Li ^[11]^	−1	−1	0	1	1	0
Wang ^[12]^	0	0	0	1	1	0
Xia et al ^[13]^	1	1	1	1	1	0
Xu and Huang ^[14]^	1	1	1	1	1	0
Hu et al ^[15]^	0	0	0	1	1	0
Zhang ^[16]^	−1	−1	0	1	1	0
Zhou and Wang ^17]^	0	0	1	1	1	0
Wang ^[18]^	0	0	0	1	1	0

1 = low risk; 0 = unclear; −1 = high risk.

### 3.3. Literature analysis results

#### 3.3.1. Clinical efficacy

**Figure 4.**
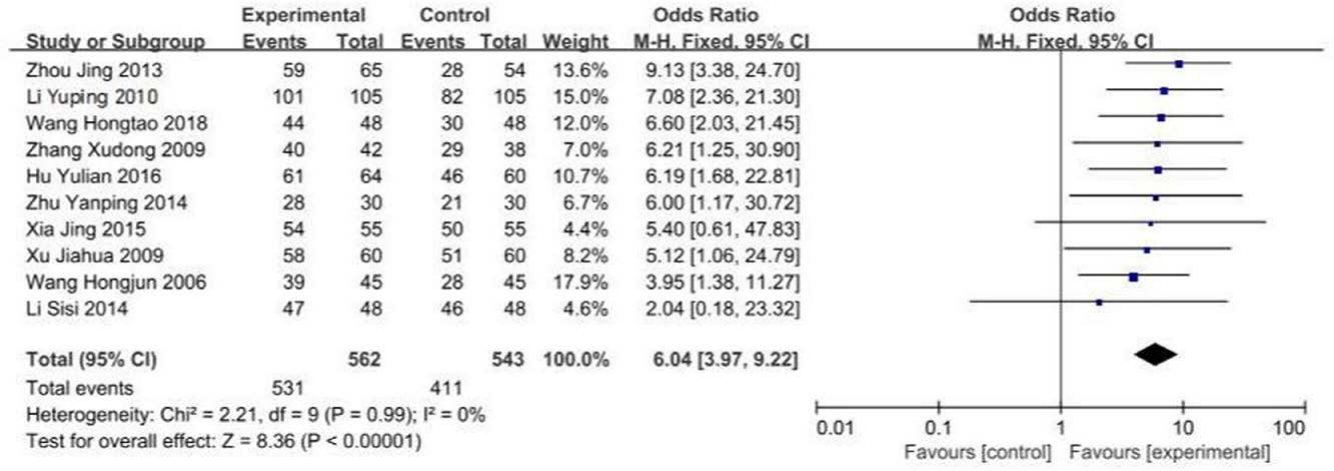
Forest plot of effective rates with low/lactose-free mill powder treated with LI patients. LI = lactose intolerance.

**Figure 5.**
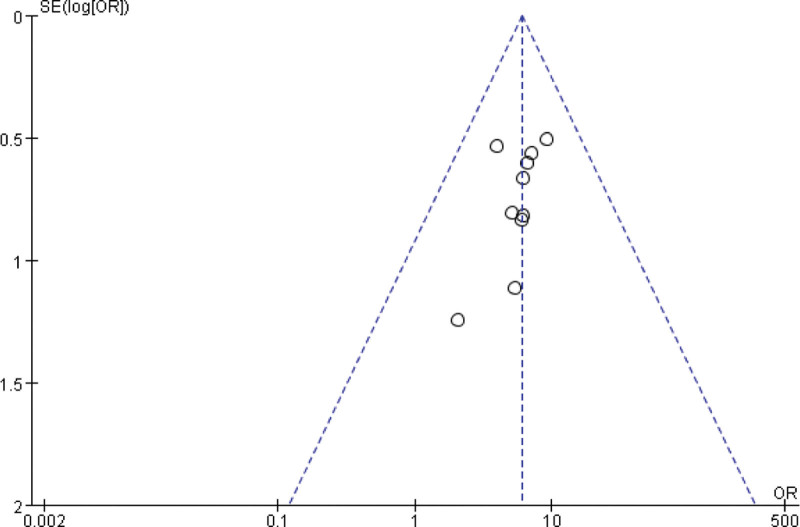
Funnel plot of the effective rate of low/lactose-free milk powder in the treatment of LI patients. LI = lactose intolerance.

All the 10 included studies reported the effective rate of low/lactose-free milk powder in the treatment of infant lactose intolerance. There was no statistical heterogeneity among the studies (*P* = .99, *I*^2^ = 0%). The fixed effect model was used. The results showed that the effective rate of low/lactose-free milk powder in the treatment group was higher than that in the control group, and the difference between the 2 groups was statistically significant (OR = 6.01, 95%Cl: 3.94–9.18, *P* < .00001; see Figs. 4 and 5).

#### 3.3.2. Analysis of treatment course

**Figure 6.**
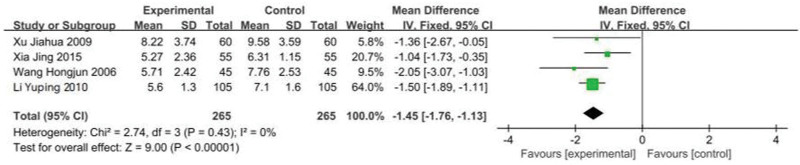
Forest plot of treatment courses in low/lactose-free milk powder in the treatment of LI patients. LI = lactose intolerance.

**Figure 7.**
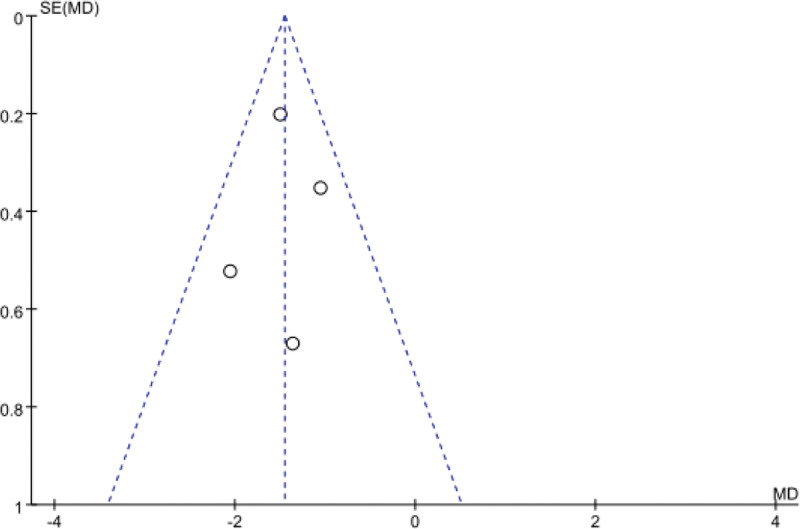
Funnel plot of treatment courses in low/lactose-free milk powder in the treatment of LI patients. LI = lactose intolerance.

Not all the 10 articles reported the comparison of treatment course after intervention measures. Meta-analysis was performed on the reported articles. There was no statistical heterogeneity among the studies (*P* = .43, *I*^2^ = 0%). The fixed effect model was used. The results showed that the incidence of treatment course in the treatment group was lower than that in the control group, and the difference between the 2 groups was statistically significant (MD = −1.45, 95%Cl: −1.76 to −1.13, *P* < .0001; see Figs. 6 and 7).

#### 3.3.3. Analysis of anti-diarrhea time

**Figure 8.**
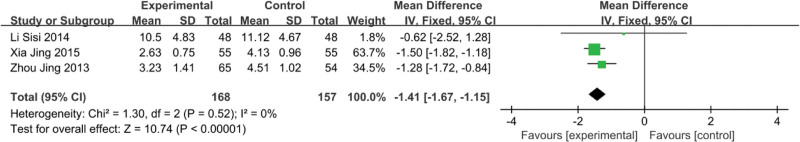
Forest plot of anti-diarrhea time in low/lactose-free milk powder in the treatment of LI patients. LI = lactose intolerance.

Not all of the 10 literatures reported the analysis of anti-diarrhea time. Meta-analysis of the literatures reporting the analysis of anti-diarrhea time showed no statistical heterogeneity among the studies (*P* = .52, *I*^2^ = 0%). The results showed that the anti-diarrhea time of infants with lactose intolerance in the treatment group was significantly shorter than that in the control group (MD = −1.41, 95%Cl: −1.67 to −1.15, *P* < .0001; see Fig. 8).

## 4. Conclusion

A total of 10 RCTs were included in this study. Meta-analysis showed that: Compared with the routine treatment group, the routine medicine combined with low/lactose-free diet treatment group could improve the clinical effective rate of infantile diarrhea (OR = 6.01, 95%Cl: 3.94–9.18, *P* < .00001), and could shorten the course of acute diarrhea; for reducing adverse reactions, improve prognosis also has excellent performance! However, lactose, as the main source of carbon water for infants and young children, is the main energy supply. If long-term low-sugar feeding can lead to malnutrition and slow development in children; attention should be paid to the nutritional intake of children.

Moreover, the number of literatures included in this analysis is not large, and the quality is also uneven, so it will have some influence on the final conclusive judgment. Only 2 of the 10 literatures report the specific method of random allocation, and the remaining literatures only indicate “random”; And in the blind allocation, only 2 articles clearly blinded, the rest of the literature did not have a specific description, at the same time, the details of the specific implementation of the treatment plan is different, the difference in the efficacy criteria will also have a certain impact on the results.

In summary, the current analysis shows that low/lactose-free diet with conventional drugs has a good effect on the treatment of diarrhea in children, which can improve the clinical efficiency of children, shorten the course of disease, and reduce the occurrence of adverse reactions; The nutrition intake of children should be paid attention to when using this protocol, because the number of original studies included is small, the quality is generally not high, and the sample size of the original studies is small, the conclusions of this study still need to be verified by more high-quality, large-sample randomized controlled original studies.

## 5. Discussion

Pediatric diarrhea is a common digestive tract disease in infants and young children. The disease can lead to the loss of nutrients in infants and young children, affecting the growth and development of children, and the disease is also one of the main causes of child death.^[19]^ There are many causes of diarrhea in children, including viral infections,^[20]^ milk-eating injury,^[21]^ complications, etc.^[22]^ Among them, rotavirus infection is a major cause of acute diarrhea, and rotavirus damage to the small intestinal mucosa is rapid and violent, resulting in weakened absorption of water by the small intestinal mucosa, and at the same time leading to lactose digestion disorders, intestinal osmotic pressure increased; Will make the body water to concentrate in the intestines, which makes a lot of water loss, causing dehydration. And for rotavirus caused by diarrhea there is no so-called specific, so speed up the treatment process, reduce the course of disease is an effective means of treatment.

And now, the main clinical treatment methods are fluid rehydration therapy, electrolyte balance, mucosal protection therapy, acupuncture and moxibustion, western medicine and so on.^[19]^ And because disaccharidase deficiency is a key factor in the rapid cure of acute diarrhea in children.^[23]^ Therefore, it is feasible and effective to carry out adjuvant treatment from this aspect. The use of low-sugar or sugar-free milk powder can shorten the course of diarrhea or lactose intolerance in children and reduce the loss of nutrients. However, lactose is an essential nutrient for the growth and development of infants and young children. Long-term consumption of low-sugar or even sugar-free foods will lead to stunting and growth retardation in children. The adjuvant treatment of diarrhea in children by low/sugar-free diet can be extended to a functional food that can regulate intestinal flora and avoid disaccharides. Fermented milk products are a good choice.

Fermented milk is a nutrient-rich food, is a good source of some beneficial trace substances, such as calcium, phosphorus, potassium, vitamin A, vitamin B2 and vitamin B12, etc, commonly used fermented dairy products are yogurt, cheese, sour cream, koumiss (fermented horse milk), etc. The various types of complex probiotic fermented milk and yogurt on the market are usually made by live bacteria (usually Lactobacillus bulgaricus and Streptococcus thermophilus) acidification and fermentation, so that the product system is more stable, shelf life and longer. In addition, fermented milk products can also provide high biological value protein and essential fatty acids. Increasing evidence shows that the consumption of yogurt and fermented milk is associated with many health benefits, including the prevention of osteoporosis, diabetes and cardiovascular disease, as well as promoting intestinal health and immune system regulation.

In this paper, we can put forward the mare’s milk fermented milk, because of its own functionality, and high nutritional value of mare’s milk, is the closest natural milk, can improve the body’s immunity, but also meet the above requirements, both by regulating the intestinal flora of acute diarrhea, and will not cause additional burden on intestinal absorption, so we can further study the future market of mare’s milk fermented milk powder. At present, the low-sugar fermentation products for medical assistance needs to be developed, I believe it will shorten the course of acute diarrhea, improve infant compliance and immunity has an excellent performance!

## Acknowledgments

We would like to thank the researchers and study participants for their contributions.

## Author contributions

**Conceptualization:** Kalibinuer Aierken, Zhiwei Xu, Jianbao Ma, Gulibahaer Kawuli.

**Data curation:** Kalibinuer Aierken, Zhiwei Xu, Jianbao Ma.

**Formal analysis:** Kalibinuer Aierken, Zhiwei Xu.

**Investigation:** Kalibinuer Aierken, Zhiwei Xu.

**Methodology:** Zhiwei Xu.

**Software:** Kalibinuer Aierken, Jianbao Ma.

**Supervision:** Kalibinuer Aierken, Gulibahaer Kawuli.

**Validation:** Kalibinuer Aierken, Gulibahaer Kawuli.

**Visualization:** Kalibinuer Aierken, Zhiwei Xu, Jianbao Ma, Gulibahaer Kawuli.

**Writing – original draft:** Kalibinuer Aierken, Zhiwei Xu.

**Writing – review & editing:** Kalibinuer Aierken, Gulibahaer Kawuli.
